# The Power of Fish Models to Elucidate Skin Cancer Pathogenesis and Impact the Discovery of New Therapeutic Opportunities

**DOI:** 10.3390/ijms19123929

**Published:** 2018-12-07

**Authors:** Sreeja Sarasamma, Yu-Heng Lai, Sung-Tzu Liang, Kechun Liu, Chung-Der Hsiao

**Affiliations:** 1 Department of Chemistry, Chung Yuan Christian University, Chung-Li 32023, Taiwan; sreejakarthik@hotmail.com; 2 Department of Bioscience Technology, Chung Yuan Christian University, Chung-Li 32023, Taiwan; stliang3@gmail.com; 3 Department of Chemistry, Chinese Culture University, Taipei 11114, Taiwan; lyh21@ulive.pccu.edu.tw; 4 Biology Institute, Qilu University of Technology (Shandong Academy of Sciences), Jinan 250103, China; 5 Taiwan Center for Biomedical Technology, Chung Yuan Christian University, Chung-Li 32023, Taiwan; 6 Center for Nanotechnology, Chung Yuan Christian University, Chung-Li 32023, Taiwan

**Keywords:** skin cancer models, zebrafish, medaka, molecule screening

## Abstract

Animal models play important roles in investigating the pathobiology of cancer, identifying relevant pathways, and developing novel therapeutic tools. Despite rapid progress in the understanding of disease mechanisms and technological advancement in drug discovery, negative trial outcomes are the most frequent incidences during a Phase III trial. Skin cancer is a potential life-threatening disease in humans and might be medically futile when tumors metastasize. This explains the low success rate of melanoma therapy amongst other malignancies. In the past decades, a number of skin cancer models in fish that showed a parallel development to the disease in humans have provided important insights into the fundamental biology of skin cancer and future treatment methods. With the diversity and breadth of advanced molecular genetic tools available in fish biology, fish skin cancer models will continue to be refined and expanded to keep pace with the rapid development of skin cancer research. This review begins with a brief introduction of molecular characteristics of skin cancers, followed by an overview of teleost models that have been used in the last decades in melanoma research. Next, we will detail the importance of the zebrafish (*Danio rerio*) animal model and other emerging fish models including platyfish (*Xiphophorus* sp.), and medaka (*Oryzias latipes*) in future cutaneous malignancy studies. The last part of this review provides the recent development and genome editing applications of skin cancer models in zebrafish and the progress in small molecule screening.

## 1. Introduction

Skin cancer, which includes squamous cell carcinoma (SCC) and melanoma, represents the most common type of cutaneous malignancy around the world and incidence rates are continuing to increase [1]. Melanoma is a major worldwide health problem due to its high prevalence and mortality rate. In 2018, the American Cancer Society estimates that approximately 91,270 people in the USA will be diagnosed with melanoma, and 9320 (about 5990 men and 3330 women) people are expected to die of melanoma skin cancer [2]. Recent studies indicated that the incidence of melanoma has increased steadily in the last three decades (available online: https://www.cancer.org/cancer/melanoma-skin-cancer/about/key-statistics.html). In a canonical model, melanoma with mutations induced by ultraviolet (UV) light may eventually lead to proliferation of melanocyte [3]. After proliferation, the first response of melanocytes are to develop into oncogene-induced senescence (OIS), which further form benign nevus [4]. Generally, UV light induces DNA damage in keratinocytes and melanocytes. Extreme UV exposure (sunburn) leads to massive death of keratinocytes, but melanocytes are able to survive due to efficient DNA damage repair response [5]. Recently, exome sequencing in melanoma with high heterogeneity has identified a large catalogue of recurrent somatic variants, which were found to be mostly within the *B-Raf proto-oncogene*, *serine/threonine kinase* (*BRAF*) and *Neuroblastoma RAS viral oncogene homolog* (*NRAS*) genes [6]. However, nearly 100% of the recurrence is due to rapidly emerging multi-drug resistance (MDR) [7]. Novel genes have not yet been discovered in human melanoma expression studies and there is a lack of known and new candidate drug targets for further validation in malignant melanoma animal model [8]. Therefore, a deep understanding of carcinogenesis in animal models is urgently needed to improve drug resistance in cancer therapy. So far, the existing melanoma animal models have been zebrafish, medaka, platyfish, the mouse, Syrian hamster, opossum and Mongolian gerbil [9,10]. Each of them has its own advantage and disadvantage in drug screening and molecular depiction of melanoma, and it will be discussed as follows.

Recently, there has been an increasing number of application of fish used as human disease models, especially in melanoma research. It is well-known that humans and fish share very few differences at the molecular level [11]. For instance, human *V-Ha-Ras Harvey Rat Sarcoma* (*HRAS)* gene is one of the most frequently mutated genes in cancer. *HRAS* shares more than 95% of similarity with the corresponding gene in medaka [12]. In biomedical research, fish has important advantages over other animal models, such as easy to breed, and able to be raised in a large number at relatively low cost. However, some aspects may diverge, such as the evolution of human chromosome 17 mapped to zebrafish Linkage group (LG) 3, 5, 12, and 15, which suggests an ancestral rearrangement [13]. Another key feature of using the fish is to combine developmental biology with the power of genetics, whereby transgenic lines can produce insightful results. Moreover, it is also feasible to perform high-throughput approaches in fish models, such as whole genome mutagenesis and chemical library drug screening [14,15]. 

## 2. Teleost Models for Melanoma Studies

In the past decades, several fish models have been established and transgenic lines have proven the idea that fish species are valuable in cancer research [16,17] (summarized in Table 1). For instance, mutagenesis screenings often identify phenotypes that are severe in human disease model [18,19]. The uncovered mutations strongly affected the functions of the gene and caused severe phenotypes. These human diseases, which are hard to clarify through analytical experiment and far from being understood, might be better understood through the evolutionary mutant models [20]. Most fish species have natural variation in genes that are corresponding to human disease, they have created genetic diversity in a similar way as in humans [19]. Genomic and transcriptomic evolutionary fish models that have been fully sequenced are summarized in Table 2. Examples of fish models for melanoma research are described in the following sections.

## 3. Zebrafish

In 1970s, George Streisinger introduced the zebrafish (*Danio rerio*) model into biomedical research laboratories. Zebrafish has been a canonical biomedical research model, which had many strains adapted for human disease studies [10]. The main reasons for the versatility of zebrafish in research with numerous experimental advantages are that it is relatively easy and economical to maintain and amenable to genetic analysis [37]. One of the key features of zebrafish as a disease model is having great power to perform high-throughput drug screening of chemical libraries [38]. These chemical screenings using embryos that harbor several disease-related phenotypes are performed in multi-wells with tens of thousands of different compounds and can lead to the identification of promising candidate molecules [29]. In addition, the zebrafish resource center ZIRC (available online: http://zebrafish.org/zirc/home/guide.php) has been established at the University of Oregon campus in Eugene, Oregon, USA, which provides a central repository for mutant, transgenic and wild-type zebrafish and distributes these resources to the research community. With their small size and short generation time, breeding and maintaining zebrafish is relatively simple and economical. In addition, researchers have established a detailed understanding of zebrafish biology and genetics to develop large numbers of techniques and powerful genetics tools, not yet available for larger mammals [39]. 

Many researchers have concluded that zebrafish is the best model for melanoma studies [28,40,41]. The nodal inhibition promotes the reversion of melanoma cells towards a melanocytic phenotype [42]. In addition, the nodal signaling plays a key role in melanoma cell plasticity and tumorigenicity in vivo [42]. The melanin biosynthesis in vertebrates depends on the function of three enzymes of the tyrosinase family, tyrosinase (Tyr), tyrosinase related protein 1 (Tyrp1), and dopachrome tautomerase (Dct or Tyrp2). The mutations in Tyr family causes melanophore death, which leads to semi-dominant phenotype in the fish [43].

A number of genes involved in melanoma formation in vivo have been identified [44]. The common master melanocyte transcription factor microphthalmia associated transcription factor (MITF) plays an important role in vertebrate melanoma formation. The temperature sensitive *mitf* zebrafish mutant can conditionally control endogenous MITF activity in vivo [45]. Studies have shown that low level of endogenous MITF activity is oncogenic with *BRAF* (V600E), which also promotes melanoma progression [45,46]. The increased telomerase activity causes many types of cancer in animal model [47]. It is found that 50–60% of melanomas formed by sunlight (UV) exposure, which causes *BRAF* mutation in vivo [48]. At the molecular level, there were very few differences between humans and the zebrafish genome. For example, the human *BRAF* gene, which is one of the most frequently mutated genes in melanoma, shares almost 96% of identity with the corresponding gene in zebrafish. Interestingly, there are very few amino acid differences in the carboxyl terminus, where the human oncogenic mutations are found**.** Nevertheless, many researchers have confirmed that the fish has the same histopathology of melanocyte in vivo [21,49]. The transgenic and gene knockout fish can be generated with appropriate techniques, such as through Tol2 transposon-mediated transgenesis and TALENs/Crispr-Cas9 mediated genome editing tools [50,51].

## 4. Downstream Signaling in BRAF/Mitogen-activated protein (MAP) Kinase Pathway

Both BRAF ^V600E^ and mutant p53 expressed in melanocytes may not always lead to melanoma in humans. However, the mutation in BRAF ^V600E^ protein results in a 700-fold increase of kinase activity over the wild-type BRAF [52]. Genotype specific amplification of protein kinase A that co-operates with BRAF and p53 mutation is suggested to be involved in melanogenesis [21,53]. The BRAF and NRAS mutations are mutually exclusive in melanomas, suggesting that mutation in single gene locus is enough to over-activate the downstream Extracellular signal-regulated kinase (ERK) pathway [54]. MITF is amplified in a subset of human melanomas, which cooperates with oncogenic BRAF ^V600E^ to transform normal melanocytes [55]. Mitf-dependent transcription is essential for melanocyte development and pigmentation. Disruption of Mitf in melanocyte or melanoma triggers profound apoptosis and is susceptible to rescue by B-cell lymphoma 2 (BCL2) overexpression [56]. Clinically, primary human melanoma expression microarrays reveals tight linkage between MITF and BCL2 [57]. Mitf is a Cyclin-dependent kinase inhibitor gene that acts as an anti-proliferative transcription factor to induce G1 arrest in the cell cycle. Moreover, cooperation between Mitf and the retinoblastoma protein Retinoblastoma 1 (Rb1) potentiates Mitf to activate transcription, which leads to dysregulation of the cell cycle and causes melanoma in humans [56]. Approximately 60% of melanomas were caused by mutations in the *BRAF* gene and resulted in an alteration of BRAF protein expression that leads to the activation of downstream signaling in the MAP kinase pathway [58]. Moreover, in 90% of the cases, the activating mutations contain the substitution of glutamic acid to valine at amino acid position 600 of the *BRAF* protein [59]. In 2011, Long and colleagues found that *BRAF* mutations were related to a high incidence of melanoma, including earlier age of onset, shortened survival, and lack of chronic skin damage, suggesting the importance of inhibiting mutated *BRAF* in a population of 197 patients [60]. Up to 25% of melanomas are associated with p53 mutations, based on studies in a zebrafish model [61]. The researchers confirmed that the ADP Ribosylation factor (ARF) deficiency leading to negative impacts on p53 activity causes melanoma in fish, as well as in humans [61,62]. It has been shown that the Telomerase reverse transcriptase (TERT) mutations caused cutaneous cancer in the animal body. The BRAF ^V600E^/p53-transgenic melanoma model in the telomerase mutant unequivocally illustrated the importance of telomere lengthening mechanisms in proliferation-associated diseases in human and zebrafish [63].

**Figure 1:**
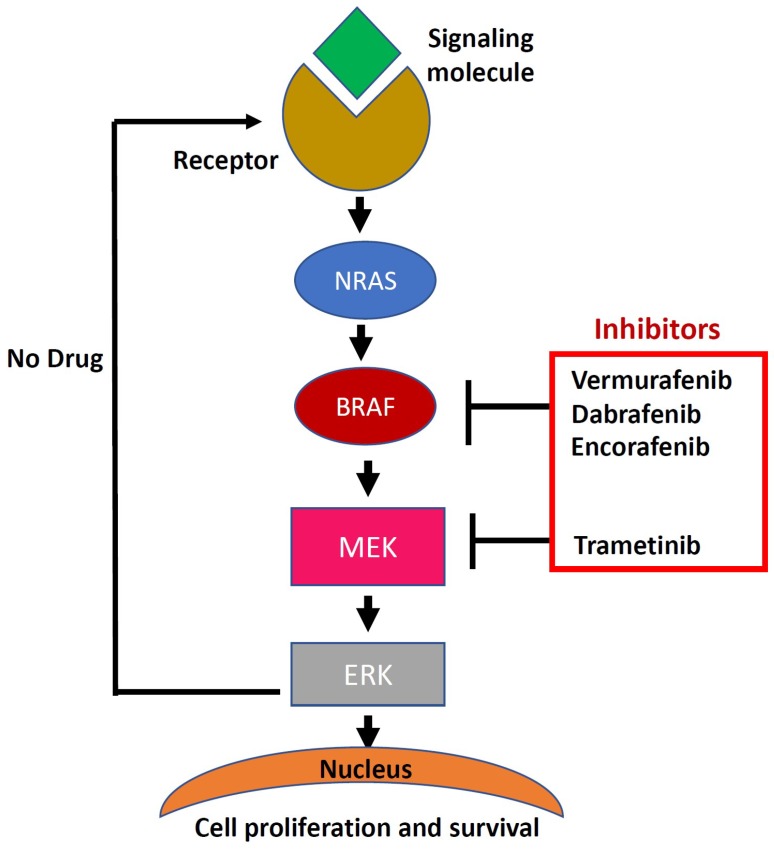
Overview of oncogenic pathways in melanoma and possibilities for targeted inhibition, relevant for the fish models described in this review. The signaling molecule binds to the receptor resulting in phosphorylation and RAS/BRAF and Mitogen-activated protein kinase (MEK) signaling to ERK, thus driving cell growth and survival. Some drugs/inhibitors can be used to block this signaling cascade and to inhibit cancer cell proliferation and survival.

Both BRAF and NRAS proteins have a significant role in a pathway called ERK or Mitogen-activated protein kinase (MAPK). These proteins are part of a mechanism that turn each protein “off” and “on” keeping cell proliferation under control [52]. Vemurafenib, the first targeted inhibitor for the BRAF mutant has been developed and approved for the treatment options for BRAF mutated metastatic melanoma. Later, in addition to vemurafenib, several selective inhibitors targeting the BRAF and MAPK pathway have been developed with promising clinical efficacies [64]. Dabrafenib and Encorafenib are the two inhibitors used to block the BRAF mutation. Figure 1 illustrates how the signaling pathways triggered by the binding of growth factors that triggers RAS/MAPK pathways leads to cell growth and proliferation.

In the last few years, the use of zebrafish model system with the advent and validation of morpholinos, as well as the increasing efficiency of transgenic techniques, has made it possible to study the effects of tens, or even hundreds of genes on specific phenotypes [65]. Specific networks, derived from the integration of expression profiling data or genetic screens in lower organisms can be validated efficiently. In addition, new hypotheses on network structure in cognate vertebrate phenotypes can be explored efficiently. Therefore, zebrafish offered a middle ground, with robust modeling of complex vertebrate biology and accessibility to functional genomics in different tissues or organs. Nonetheless, with the scalability necessary for rigorous gene–gene and gene–environment analyses, it facilitates the parallel exploration of all loci for the trait in question [66]. In a recent study, transgenic zebrafish harboring tissue-specific oncogenic alleles of human *BRAF* and *NRAS* downstream to a melanocyte-specific (*mitf*) promoter were used to manipulate zebrafish melanomas to determine the spectrum of mutations in the absence of UV light and to interrogate the role of *BRAF*, *NRAS* and *p53* in melanoma in transgenic zebrafish [22]. The results revealed that the Histone-lysine N-methyltransferase SETDB1 (SETDB1) is present in the zebrafish. SETDB1 methylates histone H3 on lysine 9 (H3K9) to accelerate melanoma formation significantly in zebrafish [67]. SETDB1 is an oncogene in melanoma and underscores the role of chromatin factors in tumorigenesis [68]. The other two histone methyltransferases (HMTs), SETDB1 and Histone-lysine N-methyltransferase SUV39H1 (SUV39H1), also cooperate with BRAF ^V600E^ to accelerate the incidence and severity of melanoma development in fish [69]. Studies shows that C-X-C motif chemokine ligand 8 (CXCL8) chemokine played an important role in B-cell lymphoma-extra large (BCL-XL) protein overexpression during tumor progression of human melanoma cells in a zebrafish xenograft model [49,70]. The transient receptor potential melastatin 7 (TRPM7) is a nonselective cation channel, which is involved in the regulation of melanocyte growth, spreading, and survival. Generally, TRPM1 and TRPM7 are expressed in human melanoma cell lines, and they are also found in zebrafish [71]. The transgenic and knockout technologies for zebrafish are well established and allow for rapid analysis of the huge amount of data related to human cancer studies. Various labs all over the world demonstrated the potential use of fish model to study functional genomics in cancer [72,73,74]. In contrast to the other models, such as mice and fly, zebrafish is unique in that its tumors biologically and morphologically mirror tumors in humans. Therefore, studying tumor microenvironment in the zebrafish model will further our understanding of cancer development.

## 5. Non-Melanoma Skin Cancer Pathogenesis in Zebrafish Model

In addition to being a valuable model in melanoma research, the fish is also a good model to study the formation and mechanisms of non-melanoma skin cancer. The fish skin model of multistage carcinogenesis continues to be a major in vivo model for studying constant and stepwise evolution of the cancer process. The initiation phase of skin carcinogenesis is facile in a teleost that involves genetic damage in forms of DNA adducts or initiator-induced DNA base changes. Compared to melanoma model, the non-melanoma skin cancer formation in fish is less reported. Scientists co-expressed active human RAC-alpha serine/threonine-protein kinase 1 (AKT1) with zebrafish smoa1-Enhanced green fluorescent protein (EGFP) fusion protein in skin and this resulted in several non-skin tumor types formation including rhabdomyoma, ocular melanoma, astrocytoma and spindle cell sarcoma [75]. Those studies suggested the oncogenic transformation threshold of the fish skin might be higher than other tissues. A group of Taiwanese scientists tried to establish a novel non-melanoma skin model in zebrafish to elucidate its role in tumorigenesis. By using Tol2 transposon, they created a transgenic zebrafish model in which different oncogene co-activation was expressed under a skin-specific *krt4* promoter [30]. They reported any single activation of either c-myc or cdc6 was unable to induce an oncogenic transformation in zebrafish skin. However, co-overexpression of transcription factor c-myc and cell cycle licensing factor cdc6 induced non-melanoma skin cancer formation at embryonic stage. This zebrafish model can be useful for initial drug screen and can also be used to predict the effects of structural modifications on chemical activity [30].

## 6. Platyfish (*Xiphophorus* Sp.)

At the beginning of the nineteenth century, the first fish melanoma model was established in *Xiphophorus.* The scientific community identified that genetic hybrids between pigmented platyfish (*Xiphophorus maculatus*) and non-pigmented swordtails (*Xiphophorus helleri*) developed spontaneous melanomas from the macromelanophors [76,77,78]. The hybrid pigments are biologically active and easily synthesized in vivo [79,80]. It was considered as a true evolutionary mutant model because the malignant skin cancers developed from naturally occurring large pigment spots that were found in several fish species with a function of kin recognition and mate choice [81]. This model was soon developed ahead of all the other evolutionary models because it offered various powerful laboratory tools [81]. The genome of platyfish was completed sequenced in 2013 and available to the public in the ENSEMBL genome server (available online: http://uswest.ensembl.org/Xiphophorus_maculatus/Info/Index). Fish cell lines have been established in a genetic stock center at the University of San Marcos (available online: http://www.xiphophorus.txstate.edu/), where inbred lines and strains derived from natural populations, as well, comprehensive information on protocols, genetic maps and referral databases are provided [82]. 

Dr. Wakamatsu has established a fish melanoma cell line named PSM1 from *Xiphophorus sp*, which can grow randomly without contact inhibition and form many clumps in vitro. High tyrosinase activity and 2-day doubling time were detected in PSM1 cell lines [83]. The histopathological analysis of malignant melanomas in the *Xiphophorus* and humans reflected the same biological phenomenon [84,85,86]. The cell lines generated from interspecific hybrids between *Xiphophorus maculatus* and *Xiphophorus helleri* have been used to evaluate the ultrastructure of pigment cells, tyrosinase activity, and the 3, 4-deoxyphenylalanine (DOPA) combined pre-melanin reactions [87]. Signaling cascades, such as the RAS/RAF/ERK1/2 pathway, the PI3K/AKT pathway, Ras-related C3 botulinum toxin substrate 1 (RAC1) and Nuclear factor kappa-light-chain-enhancer of activated B cells (NFκB)-associated pathway are involved in melanoma initiation and progression in vivo [88].

Several studies suggested that 90% of cutaneous melanoma and somatic mutations were caused by DNA damage through the exposure to UV light [89,90]. The histopathological and genetic characteristics of melanomas induced by N-methyl-N-nitrosourea (MNU) or UV radiation help to classify various types of melanoma including (i) melanocytic melanomas, (ii) melanophorous- macromelanophorous polymorphic melanomas, (iii) spindle cell type melanomas, (iv) epithelioid cell melanomas and (v) amelanotic melanomas in vivo [86]. In the case of cross and backcross, both *Xiphophorus maculatus* and *Xiphophorus helleri* showed susceptibility to developing invasive melanoma by the radiation exposure and confirmed that fish can provide similar aspects to mammalian melanoma in vivo [91,92].

In fish *Xiphophorus*, melanomas are initiated by various signaling pathways that govern tumor growth and progression [80,93,94]. Many genes important to melanoma induction, transformation, and metastasis have been identified. The alterations in oncogenes or tumor suppressor genes (*CDKN2A*, *RB1*, *Xmrk*, *Tu*, *PTEN*, *P53*) can lead to various carcinogenesis within skin [16,95,96,97]. Overexpression, duplication, or deletion of these genes can cause melanoma in fish [98,99]. On the other hand, specific compounds and nucleotides also can regulate the melanophore content in the animal body. For instance, the epinephrine causes pigment aggregation and alters the cyclic adenosine monophosphate (cAMP) content in the cells. The centrifugal and centripetal translocations of melanosomes within melanophores are regulated by the intracellular concentration of cyclic AMP in the body [100].

Kazianis et al. reported that the melanoma tumor suppressor function of controlling R-*Diff* linkage group of *Xiphophorus* fish model has structural similarity with the *Cyclin-dependent kinase inhibitor 2A* (*CDKN2)* gene in human [96]. The well-studied oncogenic *Xiphophorus* melanoma receptor kinase (Xmrk), has been characterized at the molecular level [101]. This gene loci is involved in sexual maturity, pigmentation, and melanoma formation in the fish [102,103]. The Xmrk receptor is constitutively activated with the presence of high levels of receptor molecules containing two activating mutations. These mutations are located in the extracellular domain of the receptor, and both activated *Xmrk* through the formation of disulfide-linked dimers [104]. The hypomethylation of *Xmrk* oncogenic promoter was analyzed in the *Xiphophorus* fish melanoma cell line PSM1 leading to overexpression of the *Xmrk* oncogene which caused melanoma in platyfish in vivo [105]. The microphthalmia associated transcription factor (MITF) is the main regulator of melanocyte specific gene expression, which is also involved in tyrosinase gene regulation and plays a major role in melanin synthesis pathway. The *Xmrk* suppresses the differentiation signals relayed by MITF as part of the transformation process leading to melanoma formation in the teleost [106]. Many studies have shown that this sex-linked oncogene, *Xmrk*, and an autosomal tumor suppressor gene, *Diff,* have various influences in melanoma [96]. *Xmrk* plays a major role in the formation of the pigmentation pattern in the cells; whereas *Diff* regulates proliferation in melanoma cells [107]. The esterase locus, *Est1*, is linked to the autosomal regulatory gene *Diff*, which controls differentiation of melanoma cells in the *Xiphophorus*. *Est1* locus provides a marker for monitoring the presence of the *Diff* autosome in vivo [108]. *Xmrk* oncogene encodes the receptor of tyrosine kinase related to epidermal growth factor receptor (EGFR) *in vivo* [98,109]. The melanoma-inducing EGFR-related receptor, Xmrk, specifically induces constitutive activation of Signal transducer and activator transcription 5 (STAT5) in fish melanoma cells. The direct interaction of the receptor kinase domain with the STAT protein in a phosphotyrosine-independent way led to activation of STAT5 in terms of DNA binding and target gene expression in fish [110]. The other two STAT factors, STAT1 and STAT3, implied in signaling by the Xmrk-related EGF receptor are not activated in this particular *Xiphophorus* melanoma cell type. Therefore, Xmrk initiates very specific signaling pathways and transcriptional responses in *Xiphophorus* melanoma [111]. The oncogenic EGFR variant of Xmrk induces the motility of melanocytes by interacting with focal adhesion kinase (FAK), which results in the malignant transformation of pigment cells in the system [112]. Genetic evaluation showed that the Sarcoma oncogene (src) family Src-homology 2 (SH2) domain interactions in the intracellular signaling of Xmrk have a binding affinity to the ubiquitous general receptor tyrosine kinases (RTK) substrate in fish [113]. The phosphatidylinositol 3 kinase (PI3kinase) is a substrate of Xmrk receptor, which plays a significant role in mitogenic signaling of PSM melanoma cells and the formation of malignant melanoma in *Xiphophorus* species [114]. The requirements for cooperating mutations have been confirmed in melanoma models listed in Table 1.

## 7. Medaka (*Oryzias latipes*)

Medaka is a small fresh water fish that is native to Taiwan, Japan, Korea and China. The life span of medaka in the laboratory is about 12 months and can be extended to more than 2 years. It has been widely used as a genetic model in various fields including genetics, developmental biology, evolution, and toxicology [115]. Medaka has helped to reveal many biological pathways of melanoma in vivo. The N-Methyl-N-nitro-N-nitrosoguanidine (MNNG) can induce melanoma in different strains of medaka (H04C and HB32C) [115,116,117]. To identify and decipher molecular mechanisms underlying melanoma, established medaka melanoma cells stably expressing Green fluorescence protein (GFP) have been transplanted into UV-irradiated and non-irradiated medaka, respectively. This medaka model provided an opportunity to visualize in vivo tumor cells within animal model [72]. The UV light causes alteration and progression of melanocyte resulting in melanoma or other skin-related symptoms. Studies showed that no effect of UV irradiation was observed on the expression of p53 in cell culture as well as in fish body; however, Ultraviolet-B (UV-B) radiation caused DNA damage and histological changes in the fish [118,119]. Abnormal expression of piwi interacting RNAs (piRNAs) also has been reported to associate with melanoma pathogenesis in both medaka and *Xiphophorus* fishes [120].

The oncogenic receptor tyrosine kinase, *Xmrk* is responsible for melanoma formation in vertebrates. The *Stat5* activation, *Mitf* (microphthalmia associated transcription factor) and *Bcl2* levels are correlated with the aggressive stage of the malignancy in the pigment cells [121,122]. The Xmrk is an orthologue of the human epidermal growth factor receptor (EGFR) and activates several downstream signaling pathways including direct phosphorylation of BRAF and Stat5*,* as well as the enhanced transcription of c-myc. The researchers analyzed the BRAF, Stat5 and c-myc in the medaka and confirmed that the protein motifs are highly conserved among vertebrates [123]. These results indicated that genetic factors are involved in *Xmrk* driven melanoma formation [123]. The transgenic medaka has been established as an in vivo model system for screening anticancer drugs [124]. MicroRNAs (miRNAs), a class of small noncoding RNAs, regulate gene expression at the posttranscriptional level, and is also involved in pathways leading to melanoma. Studies showed that the transgenic melanoma model in medaka is similar to human melanoma in vivo [125]. It has been shown that miR17–92 cluster (miR20a2, miR92a1, miR17 and miR18a), miR126, miR182, miR210, and miR214 are upregulated and their respective target genes (*Runt-related transcription factor 1* (*RUNX1*)*; Hypoxia-inducible factor 1A* (*HIF1A*)*; TGF-beta type 1 serine/threonine kinase receptor* (*TGFBR2*)*; Thrombospodin 1* (*THBS1*)*; and Janus kinase 2* (*JAK2*) are downregulated in both medaka and human melanoma model [126]. Recently, a group in Germany examined the whole-body transcriptome response in invasive melanoma by using transcriptomic profiling to screen for drugs in a medaka model. Their results revealed a profound down-regulation of genes involving in the immune response, particularly the innate immune system. In previous studies, novel genes have not been found yet in human melanoma; however, new candidate drug targets for further testing in this malignant melanoma medaka model have been identified [8].

## 8. Annual Fish or Killifish

Annual or killifish especially those in the genus *Limnaeus* is considered a popular model organism for studying cell cycle regulation [127]. It has an extremely fast growth rate and completes its entire life cycle within 6 weeks, which is an ideal model for aging and longevity research [128]. Killifish undergoes embryonic diapause, with embryos that can survive in dry mud for up to one year, providing a potential model for developmental diapause in vertebrates [129]. The cells of these embryos can survive in both conditions, extrinsic and intrinsic, under which it would be lethal for the majority of vertebrate cells. Mechanistic studies of this system is in its early stage; however, a number of promising discoveries suggested that this system might be a fruitful avenue that could provide novel strategies to control the cell cycle in cancer [130].

## 9. Rock Fish (*Sebastes* Sp.)

The rock fish is an ideal model system for understanding the mechanism that contributes to the speciation process due to its extensive diversity, which includes variation in morphology, ecology and a broad range of life spans [131,132,133]. Chromatophoromas are common tumors of marine and fresh water fish species which show varying degrees of differentiation with respect to specific pigment cell types [134]. Interestingly, many studies in rock fish revealed that pigmented cutaneous lesions are consistent with the cytology of human chromatophore hyperplasia and neoplasia [135].

## 10. Guppy (*Poecilia reticulata*)

Due to its high phenotypic variation, guppy is considered one of the remarkable evolutionary and ecological model organisms [136]. It was first reported by Hoffman et al. that several different sequences belonging to the middle/long-wavelength sensitive (M/L WS) and other types of opsin genes and their genetic variation existed within individuals. For more than a century, diversely colored guppy fish have attracted the attention of researchers all over the world [137,138]. Recently, a research group in the Max Plank Institute of Developmental Biology has identified three different pigment cell types in the skin of male guppies and revealed that at least two of the three types of pigment cells, xanthophores, melanophores, and iridophores, are responsible for each of the investigated ornamental traits [139].

## 11. Limitations of Animal Models in Skin Cancer Research

### 11.1. Laboratory Mice

To understand the fundamental mechanism underpinning the carcinogenesis and to develop preventive pharmaceuticals, animal experiments are inevitable for future diagnosis and treatment of cancer. Current understanding of cancer biology is demonstrated in laboratory animals due to their inbred and genetically homogeneous status. This can be genetically manipulated, allowing tissue analysis to be carried out, and enables their use as tractable models. However, complex biological experiments are neither technically nor ethically allowed in humans. Even though the mouse has been predominantly used in research for decades, questions remains focusing on its reliability as a model for human disease [140]. For instance, in the pharmaceutical industry, many drugs work very well in preclinical trials in mice, yet turn out to be ineffective when subjected to clinical trials on humans [141,142]. Moreover, there is a growing concern in the public that laboratory animals do not reflect relevant aspects of the human immune system, which may account for discrepancies on translating treatment from bench to bedsides [143,144] 

By contrast with humans, mouse skin is devoid of epidermal melanocytes as they are occupied with the hair follicles and dermis, except within ears, tails and some of the skin on footpads. In contrast, fish melanoma model mimics the appearance of melanocytes in the human epidermis. Therefore, researchers were able to establish UVB models in hairless-breed mouse to induce papilloma, blue nevi and squamous cell carcinoma, but not melanomas [145]. Studies showed that several fish species offered an excellent melanoma model with human melanocyte in the epidermis [73]. Moreover, human pigment cell tumors share conserved gene expression signatures with fish [146]. Transgenic rescue in knockout mouse is always time consuming, expensive and labor intensive. About 15% of knockouts are developmentally lethal (available online: www.genome.gov/12514551). Exclusive transgenic mouse studies were carried out only in early embryonic development. With those limitations, it is very difficult to identify genetic function related to human diseases. In addition, customized knockout mice are expensive ($3000–30,000) [140]. Memory CD8 T cells, which are thought to respond most immediately to infection, were scarce in laboratory mice and strikingly different from human adult memory CD8 T cells [143]. In contrast, CD4 and CD8 T cells are present in zebrafish [147]. The Cyclin-dependent kinase inhibitor 2A (CDKN2A) mouse model has been widely used to investigate the effect of genetic alterations in melanoma initiation, progression and metastasis [148]. However, in 1996, Serrano et al. developed a mouse strain with targeted deletion of CDKN2A locus and found that these animals developed into various malignancies but not melanoma [149]. Another model, Replication-Competent Avian sarcoma leucosis virus (ASLV) long terminal repeat with a Splice acceptor/Tumor Virus receptor A (RCAS/TVA) system also has limitations: it requires active dividing cells, and the integration is thought to be random, potentially affecting the expression of host genes [150]. Moreover, a gene with a size over 3 kilo bases cannot be successfully delivered [151]. In another study, approximately 22% of the hepatocyte growth factor/scatter factor (HGF/SF) transgenic mice developed spontaneous melanoma with a mean onset of 15 months, which does not reflect the human melanoma since they exhibited a different dermal morphology [152]. Large-scale drug screening is physically prohibitive in mice. There are several significant disadvantages of in vivo mice model in metastasis process: (1) the entire process of metastasis in a mouse model takes a long period of time; (2) evaluation of the early stage of metastasis is very difficult; (3) it is impossible to terminate and dissect mice to get real-time imaging of minute tumor lesion in deep tissues; (4) immuno-deficient mice still have residual antitumor competence that could prevent metastasis; and (5) laboratory maintenance is at a high cost throughout the experiment [153].

### 11.2. Fruitfly (*Drosophila Melanogaster*) as a Model for Skin Cancer Research

*Drosophila melanogaster* is the common fruit fly that is widely studied and highly tractable genetic model organism for underlying molecular mechanisms of human diseases. However, many types of human tumors cannot be reproduced in the fly model, particularly those related to specific tissues (breast, ovarian and prostate cancer). In addition, telomeric protection in *Drosophila* is entirely different from humans. In *Drosophila,* telomerase is maintained by transposition of specialized retrotransposons rather than by telomerase activity, and their stability is independent of the sequence of DNA termini [142]. Furthermore, the anatomy and physiology of *Drosophila* are considerably diverse from those of humans and assume only partial features of human cancer.

### 11.3. Teleost

While fish showed myriad advantages in melanoma-associated research, a viewpoint related to their post-genomic influence attracts great attention among scientists. Natural environmental impacts can also deeply diverse a phenotype, which may impede upon observation [154]. In addition, a laboratory-adjusted model system may also dilute the subtle phenomenon that plays an important role on genetic diseases [12]. Therefore, stringent and standardized laboratory protocol needs to be established to optimize the teleost model [155]. 

## 12. Cutting Edge Methods to Boost Skin Cancer Studies in Fish

In the last couple of years, the development of new sequencing technologies has enabled the rapid and cost-effective sequencing of whole genomes. By using the next-generation sequencing (NGS) methods, zebrafish mutants are now able to be mapped in weeks at less costs [11]. Whole genome sequencing (WGS) showed the potential to speed up the process of mutation detection in zebrafish. Moreover, websites Annovar (available online: http://www.openbioinformatics.org/annovar/) and Ensemble (available online: http://useast.ensembl.org/info/data/ftp/index.html) provide plenty of information that facilitates great progression in zebrafish-associated analyses. Genetic editing methods such as TALEN (transcription activator-like effector nucleases) and CRISPR-Cas9 (clustered regularly interspaced short palindromic repeats) technologies are also becoming increasingly popular in generating sequence-specific knockout or knock-in gene mutations [156,157]. TALEN for instance comprise of both TALE DNA binding domain and Fok1 cleavage domain that function to induce DNA double strand breaks at targeted sites, the breaks are then repaired through homologous or nonhomologous end-joining (NHEJ) recombination [50]. Several studies have shown a successful disruption of targeted endogenous genes in zebrafish via TALEN technology [158,159]. For example, Huang and colleagues targeted *tnikb* and *dip2a* genes that are highly expressed during embryonic developmental stage, and they were also one of the first group to demonstrate heritable TALEN-induced mutations in zebrafish [160]. Perles et al. used zebrafish to study the effect of Matrix metalloproteinase-21 (MMP21) knockout and implicated a role for MMP21 in cardiac defects and alteration of Notch signaling, which leads to asymmetric organ development [161]. Although non-viral delivery of CRISPR-Cas 9 to cells or tissues remains a key challenge, Wang and his colleagues utilized a new strategy to treat melanoma by designing single guide RNA (sgRNA) targeting Polo-like kinase-1 (PLK1) by a nano-carrier with a core of gold nanoclusters into the cell nuclei [162]. The genome editing system has had a great impact in cancer research, as it allows tissue-specific modelling of human cancer, particularly melanoma [163,164,165]

## 13. Small Molecule and Drug Screening in Zebrafish

A unique and efficient feature of the zebrafish is the potential to treat the whole organism with a drug by administering chemical compounds to the water. Although, differences in pharmacological effects between humans and zebrafish do exist, there are now hundreds of examples of small molecule screening that have conserved biological activities in humans and fish [166,167,168]. Previous studies revealed that mitf is a potential therapeutic target for skin cancer treatment [55,169]. Cheng and colleagues designed a screen of small molecule library containing 2000 compounds, and identified SKLB226 as a suppressor of mitf expression that inhibits the viability and migration of melanoma cells [170,171]. A well-known mammalian target of rapamycin (mTOR) inhibitor, rapamycin, is considered as one of the potential drugs in the field and might improve access to the kinase active site of ATP competitive inhibitors [172]. Tan et al. identified that HEXIM1 (hexamethylene bisacetamide inducible 1) knockdown by morpholino injection partially rescued *crestin* and *mitfa* expression in leflunomide (lef) treated zebrafish embryos [173]. The efficacy of cocktails of inhibitors observed by HTS (high through-put screening) in vitro, and a drug screen using rapamycin analogs combined with PIK3K/mTORi or MEKi, are currently under clinical trials [174].

## 14. Research is Changing in Advanced Melanoma (Stage III) Treatment

Recently, a controlled phase 2 trial study in which patients received neo-adjuvant therapy (given a cancer drug before surgery) to cure melanoma. Surprisingly 58% of the subjects had a complete pathologic response. The key factor of this new treatment is the *BRAF* gene mutation which present approximately 60% in melanoma patients and targeting *BRAF* gene with medication works in more than 90% of the cases [175]. Their randomized phase 2 study reveals the high-risk resectable melanoma in 23 patients with neoadjuvant nivolumb verses combined ipilimumb with nivolumab. However, treatment with combined nivolumab and ipilimumab resulted in high response rates (73%) whereas treatment with nivolumab alone yielded modest responses (25%).

## 15. Injections of Modified Herpes Virus Kills Cancer Cells

A new era of combination therapy for metastatic melanoma is now on the way. Kaufman and his colleagues demonstrated that T-VEC (Talimogen laherparepvec) is the first oncolytic virus therapy that has been effective for melanoma in a phase III clinical trial [176]. In that phase III trial, about 436 patients were randomly assigned and the overall response rate was higher in the T-VEC arm (26.4%) and resulted in a very high durabale response rate DRR (*p* < 0.001).

## 16. Summarizing Remarks

The complexity of melanoma has been well demonstrated in teleost models, which are now providing better insights to the histopathology and pathobiology of skin cancer. Regarding melanoma initiation, development and distribution, current studies suggest that fish is the best genetic as well as drug-screening model for skin cancer, particularly melanoma [16,86,177,178]. In recent years, zebrafish has become a widely used versatile model for human diseases. It has been demonstrated the zebrafish can be used as a platform for extending our understanding of molecular and cellular mechanisms, as well as develop new diagnostic and therapeutic tools. Indeed, 70% of zebrafish genes are conserved in humans, making zebrafish ideal for studying the function of human genes in cancer, cardiovascular diseases, and complex brain disorders [25,49,179]. 

Currently, drug screening relies on cell-based experiments or on animal models to confirm biological effects. The mammalian system is considered too time-consuming, expensive and complex to perform high-throughput drug screening. There is a gap between in vitro cell-based models and in vivo mammalian models. The zebrafish is an ideal model that could link preclinical toxicity screening with the drug development pipeline. Taking advantage of a highly conservative genomic, rapid development, large number of offspring, low cost and easy manipulation, the zebrafish is an excellent animal model for disease-based drug screening.

The significant progress of current research would require a full exploration and analysis to show how close these models are to the humans, in both histological and pathological aspects. This would need more comparative studies in the laboratory. In the future, we will need to focus on developmental phenotypes and to identify the pathways involved in skin cancer. We could exploit zebrafish to understand how a compound works. For instance, by analyzing the structure and functional relationship, we may apply this information in human cells. 

## Figures and Tables

**Table 1:** Development and applications of melanoma skin cancer models in zebrafish using transgenic tools.

Transgene	Key Features	References
*mitfa* : *BRAF* (*V600E*)	*BRAF* (*V600E*) expression leads to melanoma on a *tp53* mutant background	[21,22]
*mitfa* : *BRAF* (*V600E*)	Zebrafish melanoma model used for functional genetic screens to identify new oncogenes	[23]
*mitfa* : *BRAF* (*V600E*)	Genetic modulation of *mitf* leads to melanoma regression	[24]
*mitfa* : *BRAF* (*V600E*)	Zebrafish melanoma model used to screen for novel anti-melanoma drugs	[25]
*mitfa* : *NRAS* (*Q61K*)	Transgenic expression of *NRAS* (*Q61K*) leads to melanomas	[26]
*mitfa* : *HRAS* (*G12V*)	*HRAS* acts through PI3K signaling to induce melanoma	[27]
*kita* : *Gal4 x UAS* : *HRAS* (*G12V*)	*HRAS* expression in *kit*-expressing cells lead to highly penetrant and invasive phenotypes	[28,29]
*krt4: cmyc x krt4:cdc6*	Co-activation of c-myc and cdc6 in zebrafish skin to induce skin cancer formation	[30]

UAS: Upstream Activation Sequence; PI3K: Phosphoinositide 3 kinase; c-myc: cancer-myelocytomatosis.

**Table 2:** The present fish as a model completed genome sequence (the model fish genome is currently being fully sequenced).

Model Organism	Disease Model	Possibility to Maintain in Lab	Genomic Resource	Transgenic Methods	References
Zebrafish (*Danio rerio*)	Many different types of cancer	Yes	*Danio rerio (Tuebingen)*	No	[11,31]
Platy fish (*Xiphophorus maculatus*)	Cancer, specifically melanoma	Yes	Three species completed	No	[32]
Annual fish (*Cynolebias nigripinnis Nothobranchius rachovii*)	Used as a model for aging	Yes	Two species completed	Yes	[33]
Medaka fish (*Oryzias latipes)*	Used as a model most notably in toxicology	Yes, short life span and is easy to breed	One species	Yes	[34]
Rockfish (*Sebastes*)	Complete mitochondrial genome sequences	Yes, easy to rear in the laboratory	Five species	No	[35]
Guppy (*Poecilia reticulate*)	Model systems for the study of evolution, ecology, behavior, tumor genetics and genomics	Yes	One species	No	[36]
